# Causes and consequences of spatial variation in sex ratios in a declining bird species

**DOI:** 10.1111/1365-2656.12556

**Published:** 2016-07-08

**Authors:** Catriona A. Morrison, Robert A. Robinson, Jacquie A. Clark, Jennifer A. Gill

**Affiliations:** ^1^ School of Biological SciencesUniversity of East AngliaNorwich Research ParkNorwichNR4 7TJUK; ^2^ BTOThe NunneryThetfordIP24 2PUUK

**Keywords:** demography, migration, population dynamics, sex ratio, willow warbler

## Abstract

1. Male‐biased sex ratios occur in many bird species, particularly in those with small or declining populations, but the causes of these skews and their consequences for local population demography are rarely known. Within‐species variation in sex ratios can help to identify the demographic and behavioural processes associated with such biases.
2. Small populations may be more likely to have skewed sex ratios if sex differences in survival, recruitment or dispersal vary with local abundance. Analyses of species with highly variable local abundances can help to identify these mechanisms and the implications for spatial variation in demography. Many migratory bird species are currently undergoing rapid and severe declines in abundance in parts of their breeding ranges and thus have sufficient spatial variation in abundance to explore the extent of sex ratio biases, their causes and implications.
3. Using national‐scale bird ringing data for one such species (willow warbler, *Phylloscopus trochilus*), we show that sex ratios vary greatly across Britain and that male‐biased sites are more frequent in areas of low abundance, which are now widespread across much of south and east England. These sex ratio biases are sufficient to impact local productivity, as the relative number of juveniles caught at survey sites declines significantly with increasing sex ratio skew.
4. Sex differences in survival could influence this sex ratio variation, but we find little evidence for sex differences in survival increasing with sex ratio skew. In addition, sex ratios have become male‐biased over the last two decades, but there are no such trends in adult survival rates for males or females. This suggests that lower female recruitment into low abundance sites is contributing to these skews.
5. These findings suggest that male‐biased sex ratios in small and declining populations can arise through local‐scale sex differences in survival and dispersal, with females recruiting disproportionately into larger populations. Given the high level of spatial variation in population declines and abundance of many migratory bird species across Europe at present, male‐biased small populations may be increasingly common. As singing males are the primary records used in surveys of these species, and as unpaired males often sing throughout the breeding season, local sex ratio biases could also be masking the true extent of these population declines.

## Introduction

Male‐biased adult sex ratios have been shown to be common across many bird species, particularly in threatened species (Donald 2007) and in small or fragmented populations (Fretwell & Calver 1969; Dale 2001; Woolfenden, Gibbs & Sealy 2001; Zanette 2001). Variation in adult sex ratios could be driven by sex differences in demographic (e.g. mortality) and/or behavioural (e.g. dispersal) processes. For example, declines in the quality of breeding sites could lead to locally male‐biased populations through reduced likelihood of female recruitment. Alternatively, the costs of egg laying and incubation could mean that poorer food resources or greater predation pressure disproportionately impact females (Sargeant, Allen & Eberhardt 1984; Butler & Merton 1992; Post, Götmark & Murphy 2006). Male‐biased sex ratios can have important consequences for population abundance and recruitment, through impacts on operational sex ratios (Krebs & Davies 1978) and productivity. Understanding the causes and consequences of sex ratio biases can therefore be an important step in understanding the processes influencing population dynamics, but studies exploring within‐species variation in sex ratios and their consequences are rare.

Biases in adult sex ratios in birds can arise from sex biases at egg production (Komdeur 1996; Booksmythe *et al*. 2015), or from sex‐biased mortality at the nestling, juvenile or adult stages (Donald 2007; Székely *et al*. 2014). While offspring sex ratios are typically balanced, male‐biased adult sex ratios have been reported in many bird species (Donald 2007). Higher female mortality may occur as the result of disproportionately high costs associated with reproduction (Sargeant, Allen & Eberhardt 1984; Thomson, Monaghan & Furness 1998; Post, Götmark & Murphy 2006), restricted female access to high‐quality resources (Marra 2000 *;* Donald *et al*. 2007) or, for migratory species, sex differences in locations and timing of movements (Marra 2000; Newton 2006; Alves *et al*. 2013). Sex differences in the likelihood of recruitment to breeding sites may also lead to biased sex ratios, if females tend to be disproportionately attracted to areas with large numbers of conspecifics (Ward & Schlossberg 2004). Sex differences in bird populations are often estimated from capture–mark–recapture studies and, as the probability of capture during the breeding season varies between male and female individuals (Amrhein *et al*. 2012), studies which fail to account for these differences are likely to overestimate the extent of sex ratio bias (Amrhein *et al*. 2012).

Here, we use national‐scale ringing data from the British Trust for Ornithology (BTO) Constant Effort Site (CES) scheme to explore the extent, causes and consequences of sex ratio variation in breeding populations of willow warblers, *Phylloscopus trochilus,* across Britain. Willow warblers are migratory passerines that breed in Europe and winter in sub‐Saharan Africa and that recruit into the breeding population in the first year and have a typical lifespan of two years (BTO 2016). Willow warblers are recorded and caught in large numbers at survey sites throughout Britain and thus provide a good model system. In common with many other Afro‐Palaearctic migrant species at present, willow warbler abundance and population trends vary greatly across Britain, with small and declining populations in the south‐east and larger, stable or slightly increasing populations in the north‐west (Morrison *et al*. 2010; Balmer *et al*. 2013). This spatial variation in abundance provides an opportunity to explore the links between population size and sex ratios, and the associated causes and consequences of skewed sex ratios across a species range.

Using the captures and recaptures of individuals on CE sites between 1994 and 2012, and independent large‐scale census data from the national Breeding Bird Survey (BBS), we quantify (1) spatial and temporal variation in adult sex ratios across Britain, (2) the relationship between adult sex ratios and local breeding abundance, (3) the potential contribution of sex differences in adult survival rates to local sex ratios and (4) the consequences of sex biases for local productivity.

## Materials and methods

### Estimating willow warbler survival rates and sex ratios

To estimate sex‐specific annual apparent survival rates of adult willow warblers at sites across Britain, we use mark–recapture data from the BTO Constant Effort Site scheme (CES, Peach, Buckland & Baillie 1996; Peach, Baillie & Balmer 1998; Robinson, Julliard & Saracco 2009). The CES scheme uses standardized mist‐netting to monitor the abundance, breeding success and survival of common passerines in wood, scrub and wetland habitats. At each CE site, licensed ringers deploy a series of mist nets in the same positions, for the same length of time, during (usually) 12 morning and/or evening visits between May and August. We used captures (and recaptures) on CE sites to estimate the annual survival of adult willow warblers from breeding season to breeding season. In order to produce robust survival estimates, we only included sites that were operated for five or more years, were visited more than seven times during the season and had a minimum of 50 captures of males and 50 captures of females across all years. This resulted in 34 sites being selected that operated between 1994 and 2012 (Table S1, Supporting information), a period during which population trends have varied greatly across Britain (Morrison *et al*. 2010). We used only captures of adults (second calendar year or older) that had been sexed by ringers in the hand using the presence of a brood patch (female) or a cloacal protuberance (male) (Svensson 1992). On the 34 CE sites, 82% (±12% SD) of all adults were sexed on at least one capture occasion.

### Modelling adult survival

To gain robust estimates of apparent survival from this large‐scale mark–recapture data set, estimates of recapture probabilities are also required. We used a modified form of the Cormack–Jolly–Seber (CJS) formulation to estimate:

1. apparent survival probability [the probability that a marked individual alive at sampling occasion t will survive and remain in the population (i.e. not permanently emigrate) between sampling occasion *t* and *t *+* *1]. We allowed survival to vary between sites and accounted for temporal variation by including year as a random effect.
2. recapture probability (the probability that a marked individual alive and associated with the population at time *t* will be captured). We allowed recapture probabilities to vary between sexes and accounted for spatial variation by including site as a sex‐specific random effect, and we assumed constant recapture probabilities across years.

The CJS model makes a number of assumptions, which, if violated, may bias parameter estimates (Lebreton *et al*. 1992). Of particular relevance to the CES data set is the assumption that every marked individual alive in the population in a given year has the same probability of being recaptured, as individuals occupying territories further from the netting area and individuals using the site on passage are likely to have a lower likelihood of being recaught (Peach, Crick & Marchant 1995). These individuals are effectively ‘transient’ in the local population and their presence will decrease the average apparent survival probability. We therefore modified the standard CJS capture–mark–recapture model to account for the presence of transient birds by introducing an additional ‘survival period’ in the year of first capture (Hines *et al*. 2003). For each individual, we inserted an additional period after the first capture, indicating whether the bird was recaught subsequently within the same season. The probability of surviving this period can be regarded as the probability that the bird is resident on the site. The survival and recapture probabilities for this initial period are assumed to be constant across years and sites but to vary between sexes.

### Bayesian implementation of CJS survival model

We fitted a survival model to estimate survival and recapture for males and females simultaneously at each site. We used a Bayesian framework in which we combined (uninformative) priors on the parameters (adult survival, recapture probability and residency probability) with their likelihoods to calculate a posterior probability distribution (Kéry & Schaub 2012). For the sex‐specific recapture probabilities and residency probabilities, we specified Beta (1,1) priors, and for the site‐level random recapture probabilities, we specified a normal prior, with a mean of zero and variance drawn from a uniform (0,10) prior. For estimates of site‐ and sex‐specific survival, we specified uniform (0,1) priors, and we included a temporal random effect (year) with a mean of zero and variance drawn from a uniform (0,10) prior (Kéry & Schaub 2012). To summarize the posterior distribution of each parameter, we used the Markov chain Monte Carlo (MCMC) algorithm implemented in jags 3.3.0, via the r package rjags (Plummer 2003, 2013). We ran two chains of 60 000 iterations, of which we discarded the first 20 000 as ‘burn‐in’ and sampled every 10th, resulting in 6000 samples. We inspected the traceplots to ensure that the chains had converged and that there was full coverage of the appropriate parameter space.

### Estimating sex ratios

We used the sex‐specific recapture probabilities (Fig. S1) to produce weighted counts of the number of adult male and female willow warblers caught on each CE site using the following equation:

(eqn 1) $$\text{Weighted}\;{\text{count}}_{j,t,s}={\text{count}}_{j,t,s}/P_{j,s}$$

where count_*j,t,s*_ represents the total number of adult willow warblers of each sex (*j*) caught in each year (*t*) and site (*s*), and *P*_j,s_ as in eqn 1 represents the recapture probability of each sex (*j*) in each site (*s*). These weighted counts were then used to calculate the sex ratio of adults (proportion of males: males/(males + females)) at each CE site in each year. We estimated sex ratios as a derived parameter, within jags, so that the uncertainty of the estimates for capture probability was propagated into the uncertainty of the estimated sex ratio (Link & Barker 2009; Amrhein *et al*. 2012; Gelman *et al*. 2014).

To quantify annual and spatial variation in sex ratios, we fitted a Gaussian generalized linear mixed model (GLMM) using the lme4 library (Bates *et al*. 2014) in r 3.1 (R Core Development Team 2014). Sex ratio was modelled as a function of year, latitude and longitude and their interaction. We included site and year as random effects to account for the non‐independence of counts from the same site and year. We included 1/(variance of the sex ratio) as a weight in the model, to give a higher weighting to more precise estimates. We obtained *P*‐values for all GLMMs using the Satterthwaite approximation in the r library lmerTest (Kuznetsova, Brockhoff & Christensen 2014).

We estimated the mean sex ratio at each CE site by taking an average of the annual estimates of sex ratios for each iteration of the MCMC chain, within each site. We report the mean and the 95% credible interval (represented by the 0·025 and 0·975 quantiles) of the distribution of these averages.

### Estimating willow warbler abundance on CE sites

Since 1994, the abundance of common breeding bird species within the UK has been monitored as part of the BTO/Joint Nature Conservation Committee (JNCC)/Royal Society for Protection of Birds (RSPB) Breeding Bird Survey (BBS) (Newson *et al*. 2013; Harris *et al*. 2014). In the BBS, 1‐km^2^ survey squares are allocated to volunteers following a stratified random sampling procedure and coverage is representative of habitats throughout the UK. Between 1994 and 2010, the BBS covered an average of 1977 squares each year, ranging from 963 in 1994 to 3263 in 1998, providing comprehensive coverage of the majority of the UK. BBS surveyors visit each 1‐km^2^ square twice per breeding season (a minimum of four weeks apart) and record bird abundance along two parallel 1‐km transects split into 200‐m sections. Observers record all birds seen or heard, excluding birds identified as juveniles, for each 200‐m transect section.

We used the maximum of the two counts (early and late in the breeding season) to calculate the total number of willow warblers detected across all BBS transect sections within each 1‐km square for each year. We then fitted a generalized additive mixed model (GAMM) to the BBS counts as a function of latitude and longitude, in the form of a thin‐plate regression spline using the gamm() function in the mgcv library of r (Wood 2006), with a Poisson error structure and log link function. Year and site were also included as a random effect in the model to account for the non‐independence of counts from the same year and same sites. The relationship predicted by the GAMM was then used to predict the relative abundance of willow warblers at each CE site.

### Local abundance and sex ratio variation

To explore the relationship between mean sex ratio and predicted abundance, we fitted a GLM. The mean sex ratio (at each site) was fitted as a function of the predicted relative abundance (at each site). We included 1/(variance of the mean sex ratio) as a weight in the model, to give a higher weighting to more precise estimates.

### Quantifying the contribution of variation in survival rates to sex ratio bias

In order to compare apparent survival rates of male and female willow warblers at sites with differing sex ratios, we computed the correlation between male and female survival rates over the 34 sites for each MCMC iteration, giving a distribution of 6000 correlation coefficients. We report the mean and the 95% credible interval (CRI, represented by the 0·025 and 0·975 quantiles) of this distribution and the probability of a positive correlation (i.e. the proportion of correlation coefficients that are positive).

In order to explore the influence of site‐level differences in apparent survival rate on sex ratios, we calculated the expected population sex ratio given the site‐level differences in male and female survival using the following equation:

(eqn 2) $$\text{Number of males per female}=\left(\varphi_{\mathrm{male}}\left(1-\varphi_{\mathrm{female}}\right)\right)/\left(\varphi_{\mathrm{female}}\left(1-\varphi_{\mathrm{male}}\right)\right)$$

where φ_male_ = male survival and φ_female_ = female survival. We converted this value to the proportion of males (males/males + females) in order to make it directly comparable to our estimates of sex ratio calculated from the count data.

We then explored the relationship between the estimated (calculated from CES data as described above) and expected (calculated from survival estimates using eqn 2) sex ratios at each site by computing the correlation for each MCMC iteration, as described above.

### Exploring the consequences of biased sex ratios for local productivity

If male‐biased sex ratios indicate the presence of unpaired males, we would expect fewer juveniles at sites with greater male bias. To explore this, we used a Poisson GLMM to model the number of juveniles (i.e. individuals hatched that year) at each site in each year as a function of the sex ratio (proportion of males) in that year. The mean predicted willow warbler abundance (across years) at each site was also included, as a fixed factor in the model, to control for variation in numbers of juveniles with abundance (i.e. we expect sites with larger populations are more likely to have larger numbers of juveniles). Site and year were included as random effects to account for the non‐independence of counts from the same site and years.

## Results

### Spatial and temporal variation in willow warbler sex ratios

**Figure 1:**
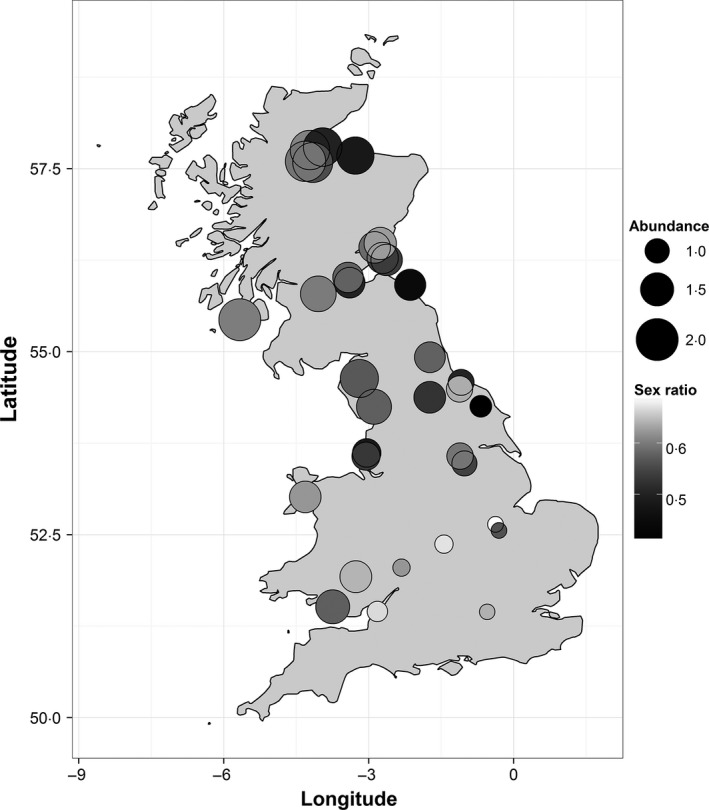
Spatial variation in the estimated sex ratio (mean proportion of males) and (log) predicted relative abundance of willow warblers on CE sites across the UK.

**Figure 2:**
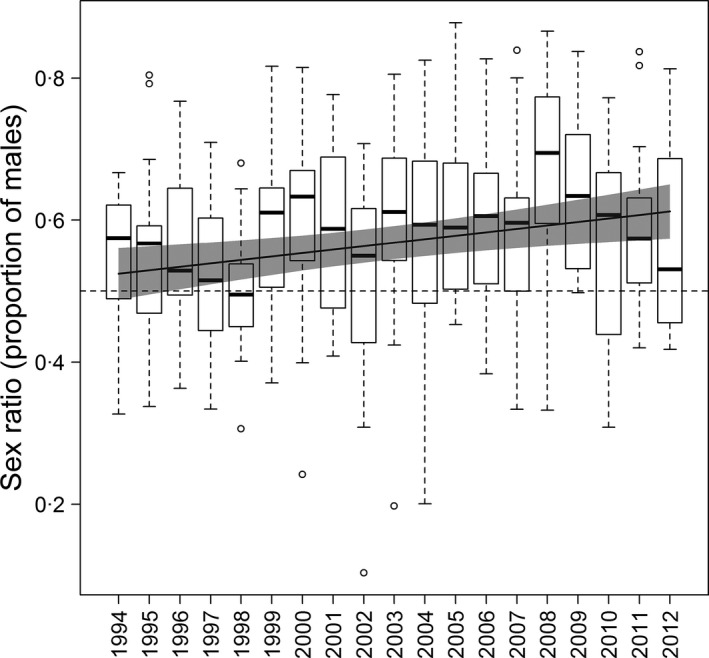
Changes in estimated sex ratios of willow warblers on CE sites. The dashed line indicates equal sex ratios and the grey line shows the predicted relationship from a GLMM (Table 1), with 95% CIs (shaded area). Annual boxplots show medians (horizontal bars), interquartile range (boxes), minimum and maximum values (whiskers) and values 1·5 times higher or lower than 1st and 3rd quartiles, respectively (circles).

Estimated adult sex ratios (proportion of males) varied markedly among CE sites (mean: 0·57 ± 0·01 SE, range: 0·44–0·68), with more male‐biased sites occurring towards the south and east of Britain (Table 1, Fig. 1). Across Britain, sex ratios have also become significantly more male‐biased over this time period; in 1994, similar proportions of males and females were estimated to occur at CE sites, but, by 2012, males comprised ~60% of the adult population on average at these sites (Fig. 2, Table 1).

**Table 1:** Results of a GLMM of the spatial and annual variation in the estimated sex ratio (proportion of males) of willow warblers on Constant Effort Sites between 1994 and 2012. Site and year were included as a random effect to control for the non‐independence of counts from sites and years. DFs calculated using the Satterthwaite formula

Fixed effects	Estimate (SE)	d.f.	*t*‐value	*P*‐value
Intercept	3·04 (0·75)	25·1	4·06	<0·001
Latitude	−0·05 (0·01)	25·4	−3·43	0·002
Longitude	0·52 (0·23)	25·6	2·31	0·02
Year	0·005 (0·001)	22·8	2·96	0·007
Latitude × Longitude	−0·01 (0·004)	25·9	−2·33	0·03

Random effects	Variance (SD)			
Site	0·002 (0·04)			
Year	0·001 (0·03)			

### Local abundance and sex ratio variation

**Figure 3:**
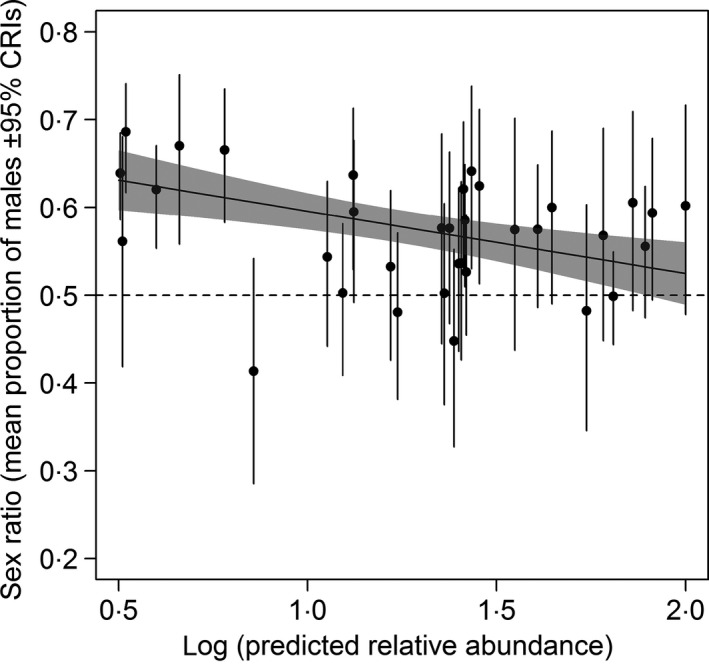
Association between sex ratio and (log) predicted relative abundance of breeding willow warblers at CE sites across the UK. The dashed line indicates equal sex ratio and the grey line shows the predicted relationship from a GLM with 95% CIs (shaded area).

Willow warbler abundance varies greatly across Britain and increases significantly from the south‐east to the north‐west (GAMM: abundance = *s*(longitude, latitude) + (1|year) + (1|site), edf = 28·6, *F* = 302·2, *P* < 0·001; Morrison *et al*. 2010). At CE sites with the highest willow warbler abundance, sex ratios were closest to equality (Fig. 3). As predicted relative abundance on CE sites declines, sex ratios become significantly more skewed, with most skewed sites being male‐biased (GLM: −0·07*x* ± 0·02SE, *t* = −3·53, *P* = 0·001, Fig. 3).

### Adult survival rates and sex ratio variation

**Figure 4:**
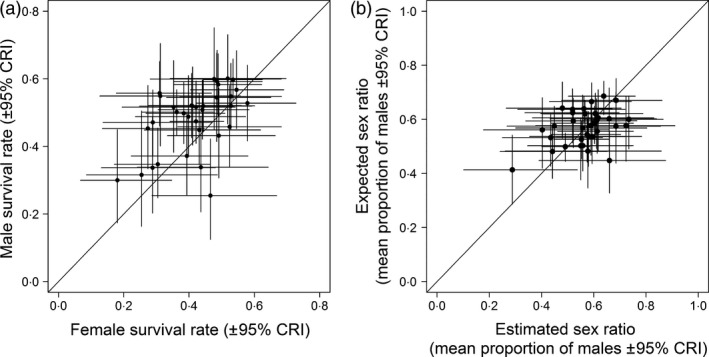
Associations between (a) male and female estimated survival rates and (b) expected (from sex differences in survival) and estimated sex ratios at CE sites. Solid lines = lines of unity.

Both male and female adult survival rates varied across the CE sites (male annual survival: 0·48 ± 0·02 SE, range: ~0·25 to ~0·60, female: 0·42 ± 0·02, range: ~0·18 to ~0·58). There was a positive correlation between male and female survival rates across sites (Fig. 4a, mean correlation coefficient: 0·35 (0·06–0·59 CRIs), probability of a positive correlation = 0·99) with lower female survival rates at the majority of sites (Fig. 4a). We found no evidence that annual variation in survival differed between males and females (Fig. S2; the variation around male and female survival rates overlaps in every year). There was a significant positive correlation between the estimated (from the count data) and expected (from the survival rates) mean sex ratios (Fig. 4b, mean correlation coefficient: 0·36 (0·06–0·62 CRIs, probability of a positive correlation = 0·99), but this correlation was dependent on one female‐biased site with a particularly large sex difference in survival [mean correlation coefficient without this site: 0·26 (−0·06 to 0·56) CRIs, probability of a positive correlation = 0·94].

### Consequences of biased sex ratios for local productivity

**Figure 5:**
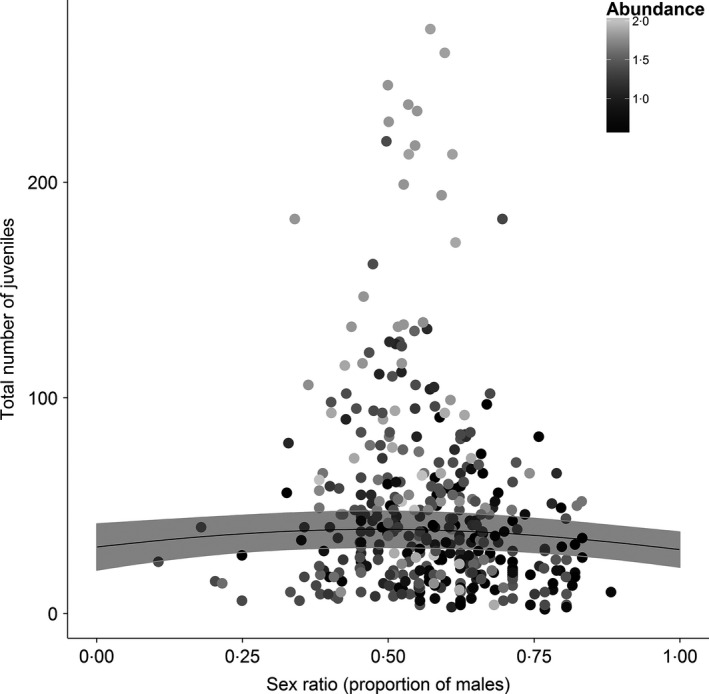
Association between the number of juveniles recorded at each CE site in each year and the estimated proportion of males. The colour of the points indicates the (log) predicted relative abundance at each site. Grey line shows the predicted relationship from a GLMM (Table 2), with 95% CIs (shaded areas).

The number of juveniles caught in CE sites provides an index of local productivity. After controlling for variation in willow warbler abundance, we found that the number of juveniles captured was highest when sex ratios were closest to equality but declined as sex ratios became more skewed (Fig. 5, Table 2). Thus, significantly fewer juveniles per adult are caught at sites with skewed sex ratios, and most of these sites have populations at very low abundance (Fig. 3).

**Table 2:** Results of a GLMM of the association between the number of juveniles and the estimated sex ratio (proportion of males) and predicted abundance of breeding birds on Constant Effort Sites between 1994 and 2012. Site and year were included as a random effect to control for the non‐independence of counts from sites and years. DFs calculated using the Satterthwaite formula

Fixed effects	Estimate (SE)	*z*‐value	*P*‐value
Intercept	2·75 (0·29)	9·45	<0·001
Sex ratio (proportion of males)	−0·39 (0·21)	−1·89	0·05
Sex ratio (proportion of males)^2^	−0·48 (0·21)	−2·29	0·02
Abundance	0·72 (0·21)	3·51	<0·001

Random effects	Variance (SD)		
Site	0·25 (0·50)		
Year	0·08 (0·30)		

## Discussion

Male‐biased sex ratios have been shown to occur frequently in small and declining populations, particularly of threatened species (Fretwell & Calver 1969; Dale 2001; Woolfenden, Gibbs & Sealy 2001; Zanette 2001; Donald 2007). Our analyses show that for the willow warbler, a species with substantial variation in local population abundance arising from differing rates of population change in recent years, male biases occur primarily in areas with small populations. In addition, the frequency of male‐biased populations is increasing, probably as a result of habitat fragmentation increasing the frequency of sites at low abundance. In Britain, population declines have been particularly severe in the south‐east regions (Balmer *et al*. 2013; Morrison *et al*. 2013), and BBS abundance estimates indicate that ~54% of 1‐km squares in the south‐east region (as defined in Morrison *et al*. 2015) have local (log) relative abundances below ~0·8, the level at which our analyses suggest male‐biased populations are particularly common (Fig. 3), while only ~1% of squares in the north‐west regions have abundance estimates below this level. This suggests that small and male‐biased populations are likely to be widespread and increasing in frequency (Figs 2 and 3).

### Causes of local sex ratio variation

Skewed adult sex ratios can arise through demographic mechanisms such as sex differences in mortality (e.g. Brooke *et al*. 2012), and/or behavioural processes such as sex‐specific patterns of recruitment to breeding sites (Nevoux *et al*. 2013). Male survival rates were typically higher than female survival rates (Fig. 4a) and, although there was a significant association between estimated sex ratios and those expected from sex differences in survival alone, this association was weak and only apparent when one site with a particularly large sex difference in survival was included (Fig. 4b). This suggests that, while higher female mortality could be occurring in low‐density, skewed populations (e.g. through processes such as greater female predation rates (Evans 2004; Nadal, Nadal & Rodrigues‐Teijeiro 1996; Post, Götmark & Murphy 2006; Grüebler *et al*. 2008) or energetic costs of breeding in fragmented habitats), this survival difference is unlikely to be sufficient to explain the widespread occurrence of skewed sex ratios, and sex differences in dispersal and recruitment are likely to also be involved.

In most bird species, males typically show greater natal philopatry than females (Pusey 1987), and higher rates of female natal dispersal could contribute to the sex ratio biases. As the great majority of low abundance sites have male‐biased populations, females may be preferentially recruiting into sites with high abundance, for example through either conspecific attraction or selection of sites with large amounts of suitable habitat. In addition, our estimates of apparent adult survival incorporate both mortality and permanent emigration, and high rates of (breeding) dispersal in females could contribute to the lower apparent survival estimates of females (Fig. 4a) and the local sex ratio variation (Fig. 1).

Although the increase in frequency of sites with biased sex ratios (Fig. 2) might suggest a divergence in male and female survival rates, we found no evidence for trends in male or female survival over the same period (Fig. S2), again suggesting that sex differences in survival are not driving the skewed sex ratios. As survival rates can only be estimated for adults and not for the first‐year recruits that will also contribute to local sex ratios (as recaptures of juveniles are rare and they cannot be sexed on morphological characteristics), it is possible that there are sex‐specific trends in survival of first‐year recruits. However, there is no obvious reason why sex differences in survival should be more apparent in the first year of life.

One further cause of apparent sex ratio bias in this data set could be due to the method by which sex is determined, for example if the cloacal protuberances of males were visible for longer during the breeding season than female brood patches, allowing a greater proportion of males to be sexed. However, as there is no reason for such a bias to vary systematically in relation to abundance or proportions of juveniles, it is very unlikely that our estimates of sex ratio were influenced by such differences.

### Consequences of sex ratio skew

Skewed adult sex ratios can potentially reduce overall productivity, if individuals are unable to find a mate and breed. The lower number of juveniles in sites with increasingly skewed sex ratios suggests that many adults (particularly males) in these sites either remain unpaired or do not breed successfully. Skewed adult sex ratios were more likely to occur in areas with low willow warbler abundance, which are more frequent in the south‐east regions. Previous studies have shown that the success of individual breeding attempts by willow warblers is also lower in the south‐east than elsewhere in Britain (Morrison *et al*. 2015), and this difference is therefore likely to be compounded by the greater frequency of unpaired males reducing numbers of nesting attempts. Allee effects of skewed sex ratios at low population densities and consequent reductions in productivity can potentially drive faster declines in small populations (Stephens & Sutherland 1999). This suggests that conservation efforts should focus on maintaining and enhancing sites capable of supporting large populations likely to have more equal sex ratios (either through greater attraction of recruiting females or higher female survival) and greater success of nesting attempts. Habitat fragmentation leading to small populations with skewed sex ratios could be an important driver of population declines in these landscapes.

### Implications for population monitoring

During the breeding season, monitoring of the abundance of many bird species (especially passerines) relies primarily on records of male song (Newson *et al*. 2013). Studies of nightingales *Luscinia megarhynchos* have shown that unpaired males sing throughout the breeding season (Amrhein *et al*. 2007) and, once mated, the singing frequency of males is considerably reduced (Catchpole 1983; Amrhein 2002). Consequently, the number of males encountered singing can be greater than the number of breeding pairs (Amrhein *et al*. 2007). As individuals singing throughout the breeding season are more likely to be detected during surveys, the reproductive capacity of populations with male‐biased sex ratios is likely to be consistently overestimated (Donald 2011). In willow warblers, 86% of records during BBS surveys are of singing males (Harris *et al*. 2014). Increases in the relative frequency of unpaired males as local population size declines, for example as a result of female avoidance of small populations and/or increased female mortality, can therefore result in systematic underestimation of population declines. If skewed sex ratios at low population size occur in the many other migratory bird species showing the same patterns of changes in abundance across Britain (Balmer *et al*. 2013; Morrison *et al*. 2013), these may be masking the true extent of their population declines.

## Data accessibility

Data associated with this study are provided in the online Supporting Information.

## Supporting information


**Figure S1.** The association between estimates of male and female recapture probability at the 34 CE sites (black line is the line of unity).
**Figure S2.** Annual variation in the random effect of year included in models of adult annual survival of male (black circles) and female (open circles) willow warblers at CE sites.
**Appendix S1.** jags code used to implement the survival model.
**Table S1.** CE Site locations and mean demographic rates.

## References

[jane12556-bib-0001] Alves, J.A. , Gunnarsson, T.G. , Hayhow, D.B. , Appleton, G.F. , Potts, P.M. , Sutherland, W.J. *et al* (2013) Costs, benefits, and fitness consequences of different migratory strategies. Ecology, 94, 11–17. 23600235 10.1890/12-0737.1

[jane12556-bib-0002] Amrhein, V. (2002) Nocturnal and diurnal singing activity in the nightingale: correlations with mating status and breeding cycle. Animal Behaviour, 64, 939–944.

[jane12556-bib-0003] Amrhein, V. , Kunc, H.P. , Schmidt, R. & Naguib, M. (2007) Temporal patterns of territory settlement and detectability in mated and unmated Nightingales *Luscinia megarhynchos* . Ibis, 149, 237–244.

[jane12556-bib-0004] Amrhein, V. , Scaar, B. , Baumann, M. & Mine, N. (2012) Estimating adult sex ratios from bird mist netting data. Methods in Ecology and Evolution, 3, 713–720.

[jane12556-bib-0005] Balmer, D.E. , Gillings, S. , Caffery, B.J. , Swann, R.L. , Downie, I.S. & Fuller, R.J. (2013) Bird Atlas 2007–11: The Breeding and Wintering Birds of Britain and Ireland. BTO Books, Thetford, UK.

[jane12556-bib-0006] Bates, D. , Maechler, M. , Bolker, B. , Walker, S. , Christensen, R.H.B. , Singmann, H. *et al* (2014) Package “Lme4”. R Foundation for Statistical Computing, Vienna, Austria.

[jane12556-bib-0501] Booksmythe, I. , Mautz, B. , Davis, J. , Nakagawa, S. & Jennions, M.D. (2015) Facultative adjustment of the offspring sex ratio and male attractiveness: a systematic review and meta‐analysis. Biological Reviews, doi:10.1111/brv.12220. 10.1111/brv.12220 26405787

[jane12556-bib-0007] Brooke, M.D.L. , Flower, T.P. , Campbell, E.M. , Mainwaring, M.C. , Davies, S. & Welbergen, J.A. (2012) Rainfall‐related population growth and adult sex ratio change in the Critically Endangered Raso lark (*Alauda razae*). Animal Conservation, 15, 466–471.

[jane12556-bib-0008] BTO . 2016 Birdfacts. Available at: http://www.bto.org/about-birds/birdfacts. [Accessed 17 May 2016].

[jane12556-bib-0009] Butler, D. & Merton, D . (1992) The Black Robin. Oxford University Press, Oxford, UK.

[jane12556-bib-0010] Catchpole, C.K. (1983) Variation in the song of the great reed warbler *Acrocephalus arundinaceus* in relation to mate attraction and territorial defence. Animal Behaviour, 31, 1217–1225.

[jane12556-bib-0011] Dale, S. (2001) Female‐biased dispersal, low female recruitment, unpaired males, and the extinction of small and isolated bird populations. Oikos, 92, 344–356.

[jane12556-bib-0012] Donald, P. (2007) Adult sex ratios in wild bird populations. Ibis, 149, 671–692.

[jane12556-bib-0013] Donald, P.F. (2011) Lonely males and low lifetime productivity in small populations. Ibis, 153, 465–467.

[jane12556-bib-0014] Donald, P.F. , Hille, S. , deBrooke, M. L. , Taylor, R. , Wells, C.E. , Bolton, M. *et al* (2007) Sexual dimorphism, niche partitioning and social dominance in the feeding ecology of the critically endangered Raso Lark *Alauda razae* . Ibis, 149, 848–852.

[jane12556-bib-0015] Evans, K.L. (2004) The potential for interactions between predation and habitat change to cause population declines of farmland birds. Ibis, 146, 1–13.

[jane12556-bib-0016] Fretwell, S.D. & Calver, J.S. (1969) On territorial behavior and other factors influencing habitat distribution in birds. Acta Biotheoretica, 19, 37–44.

[jane12556-bib-0017] Gelman, A. , Carlin, J.B. , Stern, H.S. & Rubin, D.B. (2014) Bayesian Data Analysis, Vol. 2. Chapman & Hall/CRC, Boca Raton, FL, USA.

[jane12556-bib-0018] Grüebler, M.U. , Schuler, H. , Müller, M. , Spaar, R. , Horch, P. & Naef‐Daenzer, B. (2008) Female biased mortality caused by anthropogenic nest loss contributes to population decline and adult sex ratio of a meadow bird. Biological Conservation, 141, 3040–3049.

[jane12556-bib-0019] Harris, S.J. , Risely, K. , Massimino, D. , Newson, S.E. , Eaton, M.A. , Musgrove, A.J. *et al* (2014) The Breeding Bird Survey 2013. BTO, Thetford, UK.

[jane12556-bib-0020] Hines, J.E. , Kendall, W.L. , Nichols, J.D. & Thompson, F.R. III (2003) On the use of the robust design with transient capture‐recapture models. The Auk, 120, 1151–1158.

[jane12556-bib-0021] Kéry, M. & Schaub, M . (2012) Bayesian Population Analysis Using WinBUGS: A Hierarchical Perspective. Academic Press, London, UK.

[jane12556-bib-0022] Komdeur, J. (1996) Facultative sex ratio bias in the offspring of Seychelles warblers. Proceedings of the Royal Society of London B: Biological Sciences, 263, 661–666.

[jane12556-bib-0023] Krebs, J.R. & Davies, N.B. (1978) Behavioural Ecology. *An Evolutionary Approach* Blackwell Scientific Publications, Oxford, UK.

[jane12556-bib-0024] Kuznetsova, A. , Brockhoff, P.B. & Christensen, R.H.B. (2014) LmerTest: Tests for Random and Fixed Effects for Linear Mixed Effect Models. R package, version 2.0‐3.

[jane12556-bib-0025] Lebreton, J.‐D. , Burnham, K.P. , Clobert, J. & Anderson, D.R. (1992) Modeling survival and testing biological hypotheses using marked animals: a unified approach with case studies. Ecological Monographs, 62, 67–118.

[jane12556-bib-0026] Link, W.A. & Barker, R.J. (2009) Bayesian Inference: With Ecological Applications. Academic Press, London, UK.

[jane12556-bib-0027] Marra, P.P. (2000) The role of behavioral dominance in structuring patterns of habitat occupancy in a migrant bird during the nonbreeding season. Behavioral Ecology, 11, 299–308.

[jane12556-bib-0028] Morrison, C.A. , Robinson, R.A. , Clark, J.A. & Gill, J.A. (2010) Spatial and temporal variation in population trends in a long‐distance migratory bird. Diversity and Distributions, 16, 620–627.

[jane12556-bib-0029] Morrison, C.A. , Robinson, R.A. , Clark, J.A. , Risely, K. & Gill, J.A. (2013) Recent population declines in Afro‐Palaearctic migratory birds: the influence of breeding and non‐breeding seasons. Diversity and Distributions, 19, 1051–1058.

[jane12556-bib-0030] Morrison, C.A. , Robinson, R.A. , Clark, J.A. , Leech, D.I. & Gill, J.A. (2015) Season‐long consequences of shifts in timing of breeding for productivity in Willow Warblers, *Phylloscopus trochilus* . Bird Study, 62, 1–9.

[jane12556-bib-0031] Nadal, J. , Nadal, J. & Rodrigues‐Teijeiro, J.D. (1996) Red‐legged partridge (*Alectoris rufa*) age and sex ratios in declining populations in Huesca (Spain) applied to management. Rev. Ecol (Terre Vie), 51, 243–257.

[jane12556-bib-0032] Nevoux, M. , Arlt, D. , Nicoll, M. , Jones, C. & Norris, K. (2013) The short‐and long‐term fitness consequences of natal dispersal in a wild bird population. Ecology Letters, 16, 438–445. 23360587 10.1111/ele.12060

[jane12556-bib-0033] Newson, S.E. , Massimino, D. , Johnston, A. , Baillie, S.R. & Pearce‐Higgins, J.W. (2013) Should we account for detectability in population trends? Bird Study, 60, 384–390.

[jane12556-bib-0034] Newton, I. (2006) Can conditions experienced during migration limit the population levels of birds? Journal of Ornithology, 147, 146–166.

[jane12556-bib-0035] Peach, W.J. , Baillie, S.R. & Balmer, D.E. (1998) Long‐term changes in the abundance of passerines in Britain and Ireland as measured by constant effort mist‐netting. Bird Study, 45, 257–275.

[jane12556-bib-0036] Peach, W.J. , Buckland, S.T. & Baillie, S.R. (1996) The use of constant effort mist‐netting to measure between‐year changes in the abundance and productivity of common passerines. Bird Study, 43, 142–156.

[jane12556-bib-0037] Peach, W.J. , Crick, H.Q.P. & Marchant, J.H. (1995) The demography of the decline in the British willow warbler population. Journal of Applied Statistics, 22, 905–922.

[jane12556-bib-0038] Plummer, M . (2003) JAGS: A program for analysis of Bayesian graphical models using Gibbs sampling Proceedings of the 3rd International Workshop on Distributed Statistical Computing, 125. Vienna, Austria.

[jane12556-bib-0039] Plummer, M . (2013) Rjags: Bayesian Graphical Models Using MCMC. R package version 3–10.

[jane12556-bib-0040] Post, P. , Götmark, F. & Murphy, M.T. (2006) Foraging behavior and predation risk in male and female Eurasian Blackbirds (*Turdus merula*) during the breeding season. The Auk, 123, 162–170.

[jane12556-bib-0502] Pusey, A.E. (1987) Sex‐biased dispersal and inbreeding avoidance in birds and mammals. Trends in Ecology & Evolution, 2, 295–299. 21227869 10.1016/0169-5347(87)90081-4

[jane12556-bib-0041] R Core Development Team . (2014) R: A Language and Environment for Statistical Computing. R Foundation for Statistical Computing, Vienna, Austria.

[jane12556-bib-0042] Robinson, R.A. , Julliard, R. & Saracco, J.F. (2009) Constant effort: studying avian population processes using standardised ringing. Ringing & Migration, 24, 199–204.

[jane12556-bib-0043] Sargeant, A.B. , Allen, S.H. & Eberhardt, R.T. (1984) Red fox predation on breeding ducks in midcontinent North America. Wildlife Monographs, 89, 3–41.

[jane12556-bib-0044] Stephens, P. & Sutherland, W. (1999) Consequences of the Allee effect for behaviour, ecology and conservation. Trends in Ecology & Evolution, 14, 401–405. 10481204 10.1016/s0169-5347(99)01684-5

[jane12556-bib-0045] Svensson, L. (1992) Identification Guide to European Passerines. Published privately, Stockholm, Sweden.

[jane12556-bib-0046] Székely, T. , Liker, A. , Freckleton, R.P. , Fichtel, C. & Kappeler, P.M. (2014) Sex‐biased survival predicts adult sex ratio variation in wild birds. Proceedings of the Royal Society of London B: Biological Sciences, 281, 20140342. 10.1098/rspb.2014.0342 PMC4083783 24966308

[jane12556-bib-0503] Thomson, D.L. , Monaghan, P. & Furness, R.W. (1998) The demands of incubation and avian clutch size. Biological Reviews of the Cambridge Philosophical Society, 73, 293‐304.

[jane12556-bib-0047] Ward, M.P. & Schlossberg, S. (2004) Conspecific attraction and the conservation of territorial songbirds. Conservation Biology, 18, 519–525.

[jane12556-bib-0048] Wood, S. (2006) Generalized Additive Models: An Introduction With R. Chapman & Hall, London, UK.

[jane12556-bib-0049] Woolfenden, B.E. , Gibbs, H.L. & Sealy, S.G. (2001) Demography of brown‐headed cowbirds at Delta Marsh, Manitoba. The Auk, 118, 156–166.

[jane12556-bib-0050] Zanette, L. (2001) Indicators of habitat quality and the reproductive output of a forest songbird in small and large fragments. Journal of Avian Biology, 32, 38–46.

