# Supplementary material for: Causes and consequences of spatial variation in sex ratios in a declining bird species
Source: J Anim Ecol. 2016 Jul 8;85(5):1298–306. doi: 10.1111/1365-2656.12556 (PMC5006867; doi:10.1111/1365-2656.12556)
Supplement: Supplementary file 1 — Figure S1. The association between estimates of male and female recapture probability at the 34 CE sites (black line is the line of unity). Figure S2. Annual variation in the random effect of year included in models of adult annual survival of male (black circles) and female (open circles) willow warblers at CE sites. Appendix S1. jags code used to implement the survival model. Table S1. CE Site locations and mean demographic rates. [file JANE-85-1298-s001.docx]

**Supplementary online material**

**Figure S1:** The association between estimates of male and female recapture probability at the 34 CE sites (black line is the line of unity).

**
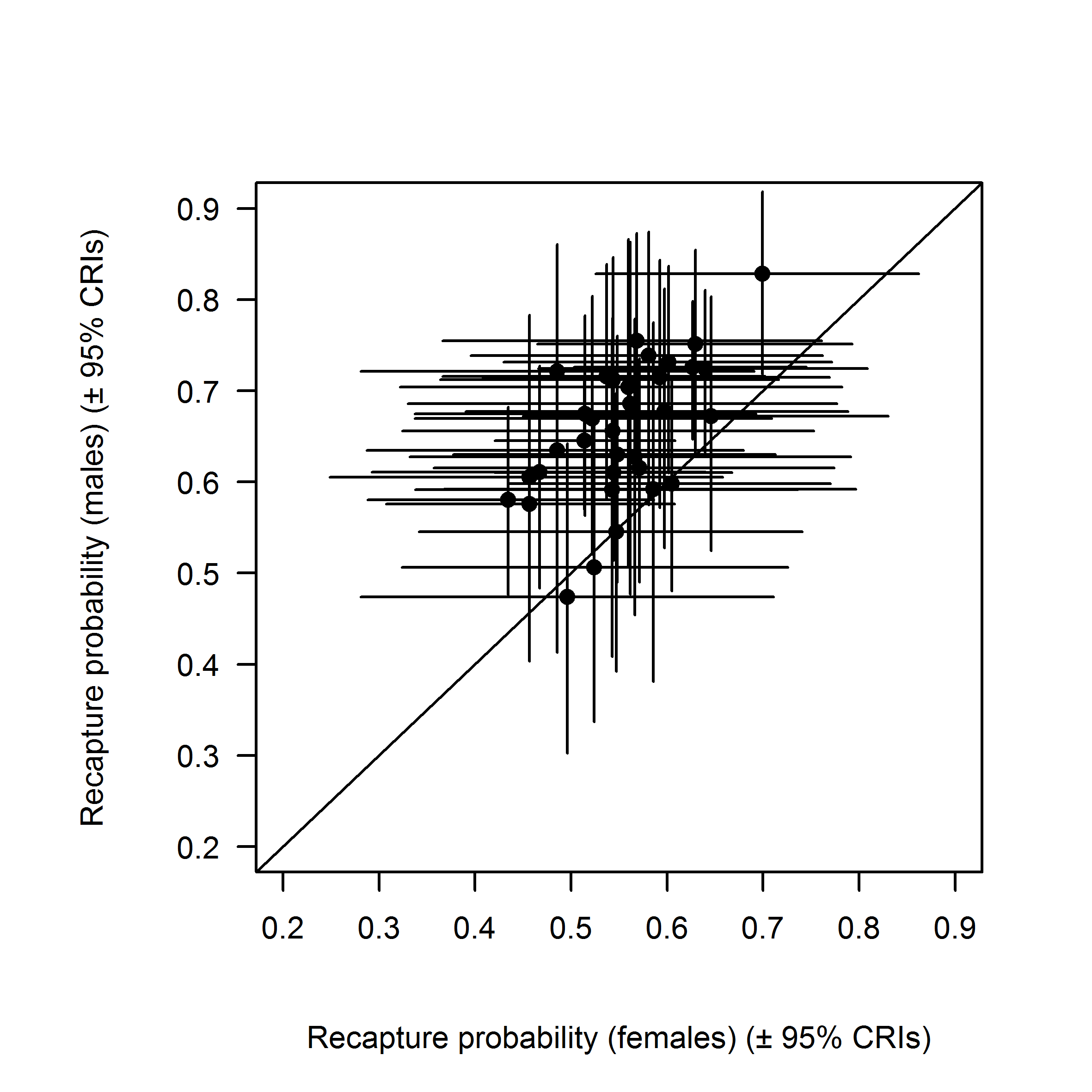
**

**Figure S2:** Annual variation in the random effect of year included in models of adult annual survival of male (black circles) and female (open circles) willow warblers at CE sites**.
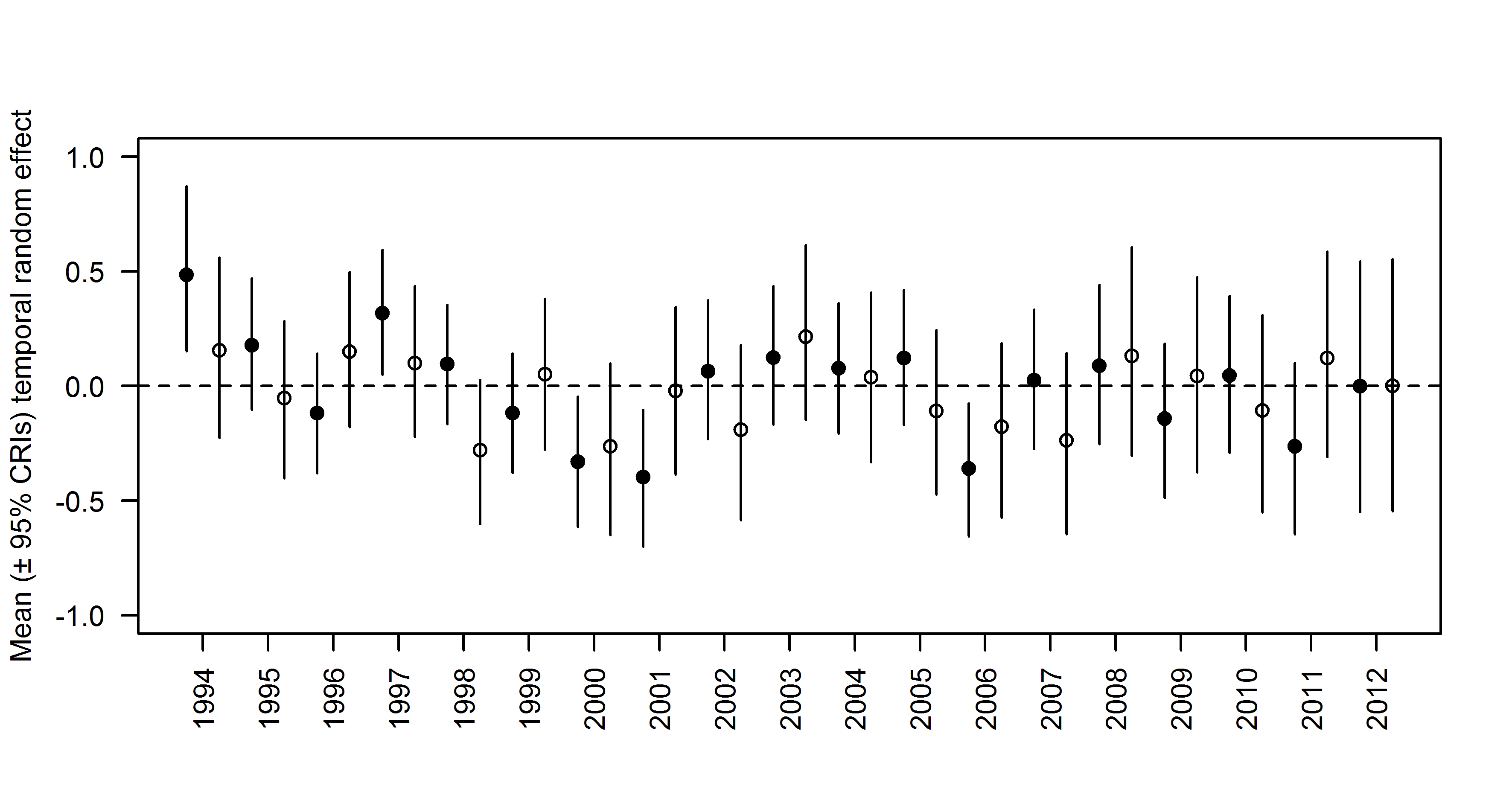
**

**Appendix S1:** JAGS code used to implement the survival model

model {

# Priors

###Survival

for (i in 1:n.individuals){

for (t in ff[i]:last.year[i]-1){

logit(phi[i,t])<-phi.g[site[i],sex[i]]+temp[t,sex[i]]

logit(p.s[i,t])<-alpha.p[sex[i]] + siteep[site[i],sex[i]]

} # t } #i

### Priors for random temporal effect on survival

sigma.t~dunif(0,10)

tau.t<-pow(sigma.t,-2)

sigma2.t<-pow(sigma.t,2)

for (ss in 1:n.sex){

for (tt in 1:nyears){

temp[tt,ss]~dnorm(0,tau.t)T(-10,10)

} #tt } #ss

### Priors for random site effect on recapture probability

sigma.s~dunif(0,10)

tau.s<-pow(sigma.s,-2) # takes variances as tau=1/var

sigma2.s<-pow(sigma.s,2)

### Priors for site effect of sex on recapture probability

for (q in 1:n.sex){

alpha.M[q] ~ dbeta(1, 1)

alpha.p[q]<-log(alpha.M[q]/(1-alpha.M[q]))

} #q

for (fs in 1:n.sites){ ##sites

for (fp in 1:n.sex){ ###sex

siteep[fs,fp] ~ dnorm(0,tau.s)T(-10,10) ##priors for sex and site specific recapture random effect

beta.p[fs,fp] ~ dnorm(0, 0.001)I(-10, 10) ##priors for within season recapture

mean.sa[fs,fp]~dunif(0,1) #### priors for mean site specific phi

phi.g[fs,fp]<-log(mean.sa[fs,fp]/(1-mean.sa[fs,fp]))

} #fp} #fs

### Prior for P(residency)

for (y in 1:n.sex){

res[y] ~ dbeta(1,1) } #y

###within season recapture probability

for (i in 1:n.individuals){ # recapture within first season depends on visit of first capture

logit(p0[i]) <- beta.p[site[i],sex[i]] * visit[i] #### 12 - visit number

} #i

# Likelihood

for (i in 1:n.individuals){

# first capture, so this = 1 by definition

z[i, ff[i]] <- 1

# First Year - within season survival(=residency) - site specific

z[i,(ff[i]+1)] ~ dbern(res[group[i]]) # State process

# Observation process, within season recapture probability in first year

yy[i,(ff[i]+1)] ~ dbern(mu0[i, ff[i]])

mu0[i, ff[i]] <- z[i, (ff[i]+1)] * p0[i]

# Now subsequent years - between year survival

for (t in ff[i]+2:last.year[i]+1){

# State process

z[i, t] ~ dbern(mu1[i, t-2])

mu1[i, t-2] <- phi[i,t-2] * z[i, t-1] ####

# Observation process

yy[i, t] ~ dbern(mu2[i, t-2])

mu2[i, t-2] <- p.s[i,t-2] * z[i, t]

} #t

} #i

###calculate sex ratio

for (xx in 1:n.sites){

recapM[xx]<- ilogit(alpha.p[1] + siteep[xx,1])

recapF[xx]<- ilogit(alpha.p[2] + siteep[xx,2])

} #xx

for(t in 1:nyears){

for (ss in 1:n.sites){

popadM[ss,t]<-nm[ss,t]/recapM[ss]

popadF[ss,t]<-nf[ss,t]/recapF[ss]

sexratio[ss,t]<-(popadM[ss,t]/(popadF[ss,t]+popadM[ss,t]))*adn[ss,t] ##adn removes the sites and years with no data

} #ss} #t

}###model

**Data:**

**Table S1:** CE Site locations and mean demographic rates.

| SiteID | Latitude | Longitude | Female survival | Male survival | log (mean relative abundance) | Sex ratio | Mean number of juveniles |
| --- | --- | --- | --- | --- | --- | --- | --- |
| 10 | 51.93 | -3.27 | 0.39 | 0.37 | 1.43 | 0.64 | 21.9 |
| 153 | 54.58 | -1.09 | 0.48 | 0.54 | 1.09 | 0.50 | 47.9 |
| 229 | 53.47 | -1.02 | 0.44 | 0.52 | 1.05 | 0.54 | 49.6 |
| 232 | 52.37 | -1.44 | 0.29 | 0.47 | 0.66 | 0.67 | 13.9 |
| 243 | 54.49 | -1.12 | 0.43 | 0.45 | 1.12 | 0.64 | 44.1 |
| 273 | 55.94 | -3.39 | 0.30 | 0.35 | 1.36 | 0.50 | 29.8 |
| 276 | 52.64 | -0.37 | 0.36 | 0.50 | 0.52 | 0.69 | 41.8 |
| 310 | 53.57 | -1.11 | 0.41 | 0.52 | 1.12 | 0.59 | 88.4 |
| 313 | 54.93 | -1.73 | 0.40 | 0.49 | 1.38 | 0.58 | 69.5 |
| 330 | 51.45 | -0.55 | 0.54 | 0.60 | 0.50 | 0.64 | 47.1 |
| 335 | 52.05 | -2.32 | 0.44 | 0.51 | 0.60 | 0.62 | 17.6 |
| 339 | 55.91 | -2.14 | 0.18 | 0.30 | 1.39 | 0.45 | 14.3 |
| 360 | 52.56 | -0.30 | 0.44 | 0.34 | 0.51 | 0.56 | 27.2 |
| 363 | 54.25 | -0.68 | 0.47 | 0.25 | 0.86 | 0.41 | 25 |
| 401 | 51.51 | -3.74 | 0.27 | 0.45 | 1.55 | 0.57 | 44.4 |
| 420 | 54.38 | -1.74 | 0.42 | 0.47 | 1.42 | 0.53 | 63.82 |
| 499 | 51.45 | -2.82 | 0.49 | 0.58 | 0.78 | 0.67 | 35.7 |
| 215 | 56.28 | -2.71 | 0.42 | 0.52 | 1.40 | 0.54 | 75.2 |
| 300 | 57.75 | -4.22 | 0.48 | 0.60 | 1.86 | 0.61 | 209.4 |
| 307 | 53.62 | -3.04 | 0.49 | 0.43 | 1.24 | 0.48 | 51.3 |
| 337 | 57.79 | -3.95 | 0.53 | 0.52 | 1.81 | 0.50 | 174.2 |
| 354 | 55.79 | -4.04 | 0.31 | 0.56 | 1.65 | 0.60 | 59.5 |
| 359 | 56.01 | -3.43 | 0.31 | 0.55 | 1.35 | 0.58 | 88 |
| 377 | 55.44 | -5.67 | 0.35 | 0.52 | 2.00 | 0.60 | 46.6 |
| 381 | 56.25 | -2.63 | 0.52 | 0.60 | 1.41 | 0.54 | 24.6 |
| 384 | 56.42 | -2.88 | 0.49 | 0.59 | 1.42 | 0.59 | 43.3 |
| 389 | 54.63 | -3.19 | 0.29 | 0.34 | 1.78 | 0.57 | 56.5 |
| 403 | 57.58 | -4.16 | 0.38 | 0.50 | 1.89 | 0.56 | 88.8 |
| 601 | 54.25 | -2.89 | 0.58 | 0.53 | 1.61 | 0.57 | 25.8 |
| 735 | 53.57 | -3.06 | 0.52 | 0.46 | 1.22 | 0.53 | 14.8 |
| 741 | 53.02 | -4.31 | 0.41 | 0.52 | 1.41 | 0.62 | 47 |
| 744 | 57.60 | -4.31 | 0.55 | 0.57 | 1.91 | 0.59 | 32.4 |
| 754 | 56.48 | -2.76 | 0.53 | 0.55 | 1.45 | 0.62 | 40.3 |
| 770 | 57.68 | -3.27 | 0.25 | 0.32 | 1.74 | 0.48 | 46.8 |
